# Optimizing Maximal Fat Oxidation Assessment by a Treadmill-Based Graded Exercise Protocol: When Should the Test End?

**DOI:** 10.3389/fphys.2019.00909

**Published:** 2019-07-23

**Authors:** Francisco J. Amaro-Gahete, Guillermo Sanchez-Delgado, Jørn W. Helge, Jonatan R. Ruiz

**Affiliations:** ^1^ EFFECTS-262 Research Group, Department of Medical Physiology, School of Medicine, University of Granada, Granada, Spain; ^2^ PROmoting FITness and Health Through Physical Activity Research Group (PROFITH), Department of Physical Education and Sports, Faculty of Sport Sciences, University of Granada, Granada, Spain; ^3^ Xlab, Center for Healthy Aging, Department of Biomedical Sciences, Faculty of Health Sciences, University of Copenhagen, Copenhagen, Denmark

**Keywords:** maximal fat oxidation, Fatmax, peak fat oxidation, methodology, RER

## Abstract

Maximal fat oxidation during exercise (MFO) and the exercise intensity eliciting MFO (Fatmax) are considered important factors related to metabolic health and performance. Numerous MFO and Fatmax data collection and analysis approaches have been applied, which may have influenced their estimation during an incremental graded exercise protocol. Despite the heterogeneity of protocols used, all studies consistently stopped the MFO and Fatmax test when the respiratory exchange ratio (RER) was 1.0. It remains unknown however whether reaching a RER of 1.0 is required to have an accurate, reliable, and valid measure of MFO and Fatmax. We aimed to investigate the RER at which MFO and Fatmax occurred in sedentary and trained healthy adults. A total of 166 sedentary adults aged between 18 and 65 years participated in the study. MFO and Fatmax were calculated by an incremental graded exercise protocol before and after two exercise-based interventions. Our findings suggest that a graded exercise protocol aiming to determine MFO and Fatmax could end when a RER = 0.93 is reached in sedentary healthy adults, and when a RER = 0.90 is reached in trained adults independently of sex, age, body weight status, or the Fatmax data analysis approach. In conclusion, we suggest reducing the RER from 1.0 to 0.95 to be sure that MFO is reached in outliers. This methodological consideration has important clinical implications, since it would allow to apply smaller workload increments and/or to extend the stage duration to attain the steady state, without increasing the test duration.

## Introduction

Metabolic flexibility is defined as the capacity of an individual to respond or adapt the nutrient balance to different metabolic demands (Goodpaster and Sparks, 2017). This concept has been particularly studied in the fasted state as well as in the shift from fasting to fed (Galgani et al., 2008). The maximal fat oxidation during exercise (MFO) and the intensity that elicit MFO (Fatmax) are key factors of metabolic flexibility (Goodpaster and Sparks, 2017; Maunder et al., 2018), affecting both endurance performance and metabolic health (Goodpaster and Sparks, 2017; Maunder et al., 2018), therefore their accurate determination is of clinical interest.

In 2002, Achten et al. (2002) were the first to validate a graded exercise protocol to determine MFO and Fatmax using 3-min duration stages and 35-W workload increments. Later, other graded exercise protocols to determine MFO and Fatmax have been applied considering participants’ sex, age, training status, or body weight status (Amaro-Gahete et al., 2019c). Two specific issues have traditionally been modified on the graded exercise protocol: (1) the stage duration (e.g., from 1 to 10 min) and (2) the workload increment (e.g., from 10 to 50 W) (Amaro-Gahete et al., 2019c).

There is no consensus regarding the ideal stage duration of a graded exercise protocol for determining MFO and Fatmax (Amaro-Gahete et al., 2019c), yet reaching the steady state seems mandatory (Shephard and Aoyagi, 2012; MacFarlane, 2017). Achten et al. (2002) showed no differences in MFO and Fatmax between 3 and 5-min stage protocols in moderately trained men (Achten et al., 2002), whereas others reported that 3-min stage duration is not long enough to reach a steady state in obese and diabetic patients with very low maximal oxygen uptake (VO2max) levels, and recommended 6-min stage duration in sedentary patients (Bordenave et al., 2007; Brun et al., 2011).

The workload increment has also largely varied across studies and, alike the stage duration, it has been adjusted to the participant’s biological characteristics (Maunder et al., 2018; Amaro-Gahete et al., 2019c). Applying relatively small workload increments allows to accurately determine MFO and Fatmax, independently of the participant’s biological characteristics.

Taking into account the above-mentioned issues, it would be advisable to apply long stage duration to reach the steady state (e.g., 6-min stage duration for sedentary patients with low levels of VO2max), and also to select small workload increments (e.g., 10-W increments) to accurately determine MFO and Fatmax through a graded exercise protocol. However, this can result in a very long test duration, which could negatively influence determination of MFO and Fatmax due to peripheral and/or central fatigue (Hultman and Greenhaff, 1991). Therefore, the development of strategies aiming to decrease the total duration of a graded exercise protocol, while using long enough stage durations and relatively small workload increments, is of clinical relevance.

Of note is that despite the heterogeneity of protocols used, all studies consistently stopped the MFO and Fatmax test when the respiratory exchange ratio (RER) was 1.0. This criterion was first applied by Achten et al. (2002) and all the subsequent studies followed the same criteria. It remains unknown however whether reaching a RER of 1 is required to have an accurate, reliable, and valid measure of MFO and Fatmax in both sedentary and trained individuals. We aimed to investigate the RER at which MFO and Fatmax occurred in sedentary and trained healthy adults.

## Materials and Methods

### Participants

This is a retrospective study of 124 young sedentary adults (age: 22.1 ± 2.2 years; body mass index: 25.0 ± 4.8 kg/m^2^; maximal oxygen uptake: 41.2 ± 7.8 ml/kg/min; 83 women/41 men) (Sanchez-Delgado et al., 2015) and 42 middle-aged sedentary adults (age: 52.1 ± 4.6 years; body mass index: 27.8 ± 3.6 kg/m^2^; maximal oxygen uptake: 30.4 ± 5.6 ml/kg/min; 23 women/19 men) (Amaro-Gahete et al., 2018a) were included in the current study. Both cohorts completed two different exercise-based interventions (24 and 12 weeks, respectively) and a total of 52 young trained adults (age: 22.6 ± 2.2 years; body mass index: 24.3 ± 5.1 kg/m^2^; maximal oxygen uptake: 44.3 ± 9.5 ml/kg/min; 35 women/17 men) and 30 middle-aged trained adults (age: 52.4 ± 4.6 years; body mass index: 27.1 ± 3.9 kg/m^2^; maximal oxygen uptake: 34.8 ± 6.3 ml/kg/min; 15 women/15 men) finished their respective exercise training programs. Before participating in this study, the participants signed an informed consent form. The investigations were approved by the Human Research Ethics Committee of the University of Granada (no. 924), and by the Human Research Ethics Committee of the Junta de Andalucía (no. 0838-N-2017).

### Procedures

**Figure 1:**
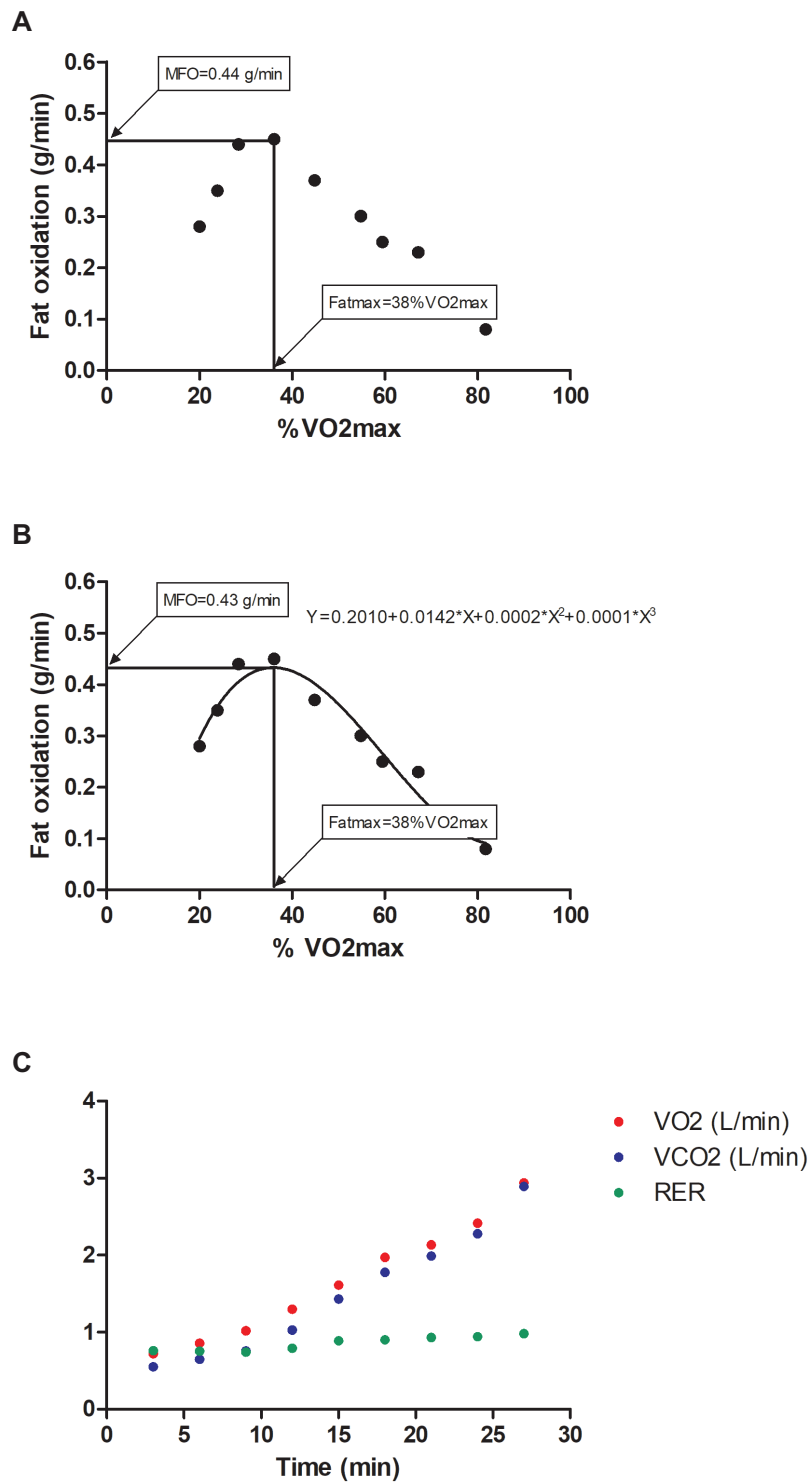
Case study example of a single participant. **(A)** It shows maximal fat oxidation during exercise (MFO) and the intensity that elicit MFO (Fatmax) using the measured-values data analysis approach (i.e., the highest fat oxidation rate recorded across the graded exercise protocol). **(B)** It shows MFO and Fatmax building a 3rd polynomial curve with intersection at (0,0) from a graphical depiction of fat oxidation data as a function of exercise intensity expressed as percentage of the maximal oxygen uptake. **(C)** It shows the single participant response to VO2/VCO2 exchange and respiratory exchange ratio during the graded exercise protocol.

We assessed MFO and Fatmax through a walking graded exercise protocol (Amaro-Gahete et al., 2018b, 2019a) before and after both exercise training programs. Participants were instructed to avoid any vigorous or moderate physical activity (48 and 24 h, respectively) before the testing day. They were asked not to consume stimulant beverages or dietary supplements during the 24 h before to test. Participants came to the research center in a fasting state of 6–7 h (~6.2 h) avoiding any type of physical activity. The graded exercise protocol began with a 3-min warm-up at 3.5 km/h with a gradient of 0% followed by increments of the treadmill speed of 1 km/h every 3 min until the maximal walking speed (previously determined) was reached. Afterward, the treadmill speed was maintained and the treadmill gradient increased 2% every 3 min until RER reached 1.0 (Jeukendrup and Wallis, 2005). Gas exchange parameters in the submaximal test were averaged every 10 s with the Breeze Suite software (version 8.1.0.54 SP7, MGC Diagnostic®). We considered the last 1 min of each 3-min stage (Amaro-Gahete et al., 2019b) to calculate fat oxidation (g/min) using the Frayn stoichiometric equation (Frayn, 1983). We determined MFO and Fatmax using the measured-values data analysis approach (i.e., the highest fat oxidation rate recorded across the graded exercise protocol; Figure 1A) and building a 3rd polynomial curve with intersection at (0,0) from a graphical depiction of fat oxidation data as a function of exercise intensity expressed as percentage of the maximal oxygen uptake (Figure 1B). A single participant response to VO2/VCO2 exchange and RER during the graded exercise protocol can be seen in Figure 1C.

## Results

### Respiratory Exchange Ratio at Maximal Fat Oxidation in Sedentary Healthy Adults

**Figure 2:**
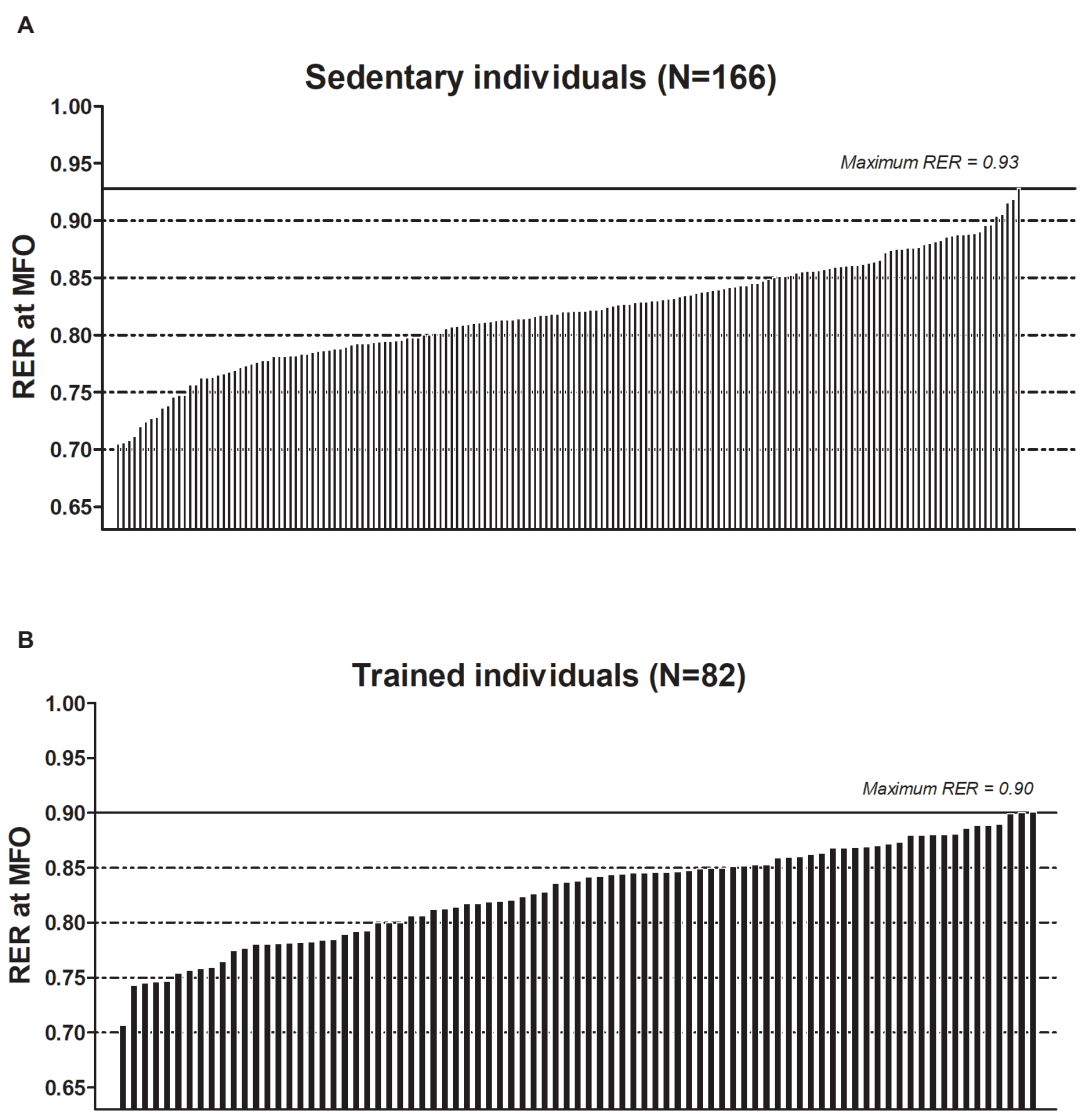
Respiratory exchange ratio (RER) reached at the intensity that elicits the maximal fat oxidation during exercise (Fatmax) **(A)** in 124 young sedentary adults (83 women/41 men) and in 42 middle-aged sedentary adults (23 women/19 men) and **(B)** in 52 young sedentary adults (35 women/17 men) and in 30 middle-aged sedentary adults (15 women/15 men).

We observed a RER at MFO of 0.82 ± 0.04 (range: 0.70–0.93; Figure 2A), which was similar in men and women (0.83 ± 0.05 vs. 0.82 ± 0.04, respectively, *p* > 0.9), in young and middle-aged adults (0.83 ± 0.05 vs. 0.82 ± 0.05, respectively, *p* > 0.8), and across body weight status (0.82 ± 0.03, 0.82 ± 0.05 and 0.83 ± 0.05 for normal-weight, overweight, and obese individuals, respectively, *p* > 0.8). Interestingly, the RER at MFO were between 0.7 and 0.8 in 34.7% (*n* = 58) of participants, between 0.8 and 0.9 in 62.3% (*n* = 104) of participants, and between 0.9 and 0.93 in 3% (*n* = 5) of participants. To note is that the graded exercise protocol total duration was 21.3 ± 4.7 min when RER = 1.0, while if the graded exercise protocol had been stopped at the highest registered RER at MFO (i.e., 0.93), the total duration would have been 13.4 ± 5.3 min (mean difference: 7.9 ± 2.9 min).

### Respiratory Exchange Ratio at Maximal Fat Oxidation in Trained Healthy Adults

The RER at MFO was 0.82 ± 0.06 (range: 0.67–0.90; Figure 2B). As in the sedentary group, we observed no sex (0.84 ± 0.05 vs. 0.81 ± 0.06, men and women, respectively, *p* = 0.3), age (0.83 ± 0.04 vs. 0.82 ± 0.05, young and middle-aged adults, respectively, *p* > 0.9) and body weight status (0.81 ± 0.06, 0.82 ± 0.06, 0.82 ± 0.05, for normal-weight, overweight, and obese individuals, respectively, *p* > 0.9) differences. The RER at MFO was between 0.67 and 0.7 in 5.7% (*n* = 5) of participants, between 0.7 and 0.8 in 26.1% (*n* = 23) of participants, and between a 0.8 and 0.9 in 68.2% (*n* = 60) of participants. To note is that the graded exercise protocol total duration was 24.0 ± 4.6 min when RER = 1.0, while if we had considered that the graded exercise protocol ended at the highest registered RER at MFO (i.e., 0.9), the total duration would have been 15.6 ± 5.8 min (mean difference 8.5 ± 3.7 min).

## Discussion

Taken together, these findings suggest that a graded exercise protocol aiming to determine MFO and Fatmax could end when a RER = 0.93 is reached in sedentary healthy adults, and when a RER = 0.90 is reached in trained adults independently of sex, age, and body weight status in walking graded exercise protocols. Whereas these figures should be confirmed in other studies, we suggest reducing the RER from 1.0 to 0.95 to be sure that MFO is reached in outliers individuals.

More sophisticated data analysis approaches, such as 2nd or 3rd polynomial curve with intersection in (0,0) have been applied to accurately estimate MFO and Fatmax (Stisen et al., 2006; Croci et al., 2014). These methodologies require at least four fat oxidation values (preferably six or more) to determine MFO and Fatmax. Reducing the maximum RER from 1.0 to 0.95 could lead to fewer fat oxidation points, and may hamper the application of those methods. To this end, we used the baseline data of the above-mentioned cohorts to calculate MFO by a 3rd polynomial curve using all fat oxidation values when RER was ≤0.95 and when RER ≤1.0. No meaningful differences in MFO were observed between both methodologies (0.37 ± 0.12 vs. 0.36 ± 0.11 g/min, for RER ≤ 1.0 and RER ≤ 0.95 respectively; *p* = 0.971). Similarly, there were no differences in MFO calculated with the measured-values data analysis approach (0.34 ± 0.11 vs. 0.34 ± 0.12 g/min, for RER ≤ 1.0 and RER ≤ 0.95 respectively; *p* = 0.924). These findings suggest that reducing maximum RER to 0.95 does not affect the MFO estimation. Reducing maximum RER until 0.95 would allow to apply smaller workload increments without increasing the test duration, which would allow more fat oxidation values around Fatmax, increasing the accuracy of the MFO estimation.

### Limitations

Our data should however be taken with caution since we conducted a treadmill test, and we do not know whether these findings can be extended to cycle ergometer test. Of note is also that our participants were healthy adults, thus future studies are needed to elucidate if these results can be applied to younger people or to patients. Future studies should confirm these findings in other populations of elite athletes or very well-trained individuals. Moreover, future studies are needed to describe the slow component effect on VO2 kinetics in graded exercise protocols aiming to determine MFO and Fatmax. Finally, the work rates of our graded exercise protocol were based on absolute increments of the treadmill grade, instead of a personalized workload increase (i.e., % of VO2max).

## Conclusion

In summary, our results have important implications, and may allow to substantially reduce the graded exercise protocol duration to assess MFO and Fatmax. Further studies are needed to investigate the impact of reducing the RER criteria on the MFO and Fatmax accuracy, by means of increasing the stage duration to attain the steady state and decreasing the workload increments magnitude.

## Data Availability

The raw data supporting the conclusions of this manuscript will be made available by the authors, without undue reservation, to any qualified researcher.

## Ethics Statement

The investigations were approved by the Human Research Ethics Committee of the University of Granada (No. 924), and by the Human Research Ethics Committee of the Junta de Andalucía (No. 0838-N-2017).

## Author Contributions

FA-G drafted the article. FA-G, GS-D, JH, and JR fully reviewed and criticized the original article. FA-G, GS-D, JH, and JR reviewed and approved the final manuscript.

### Conflict of Interest Statement

The authors declare that the research was conducted in the absence of any commercial or financial relationships that could be construed as a potential conflict of interest.
